# Validity of a simplified screening instrument for assessing overweight children in a dental setting: a cross sectional study

**DOI:** 10.1186/s12887-017-0808-x

**Published:** 2017-02-17

**Authors:** Amir Azarpazhooh, Anoushe Sekhavat, Michael J. Sigal

**Affiliations:** 1 Faculty of Dentistry, University of Toronto, 710F-481 University Ave, Toronto, ON M5G 2P1 Canada; 2 Department of Dentistry, Mount Sinai Hospital, Toronto, Canada; 3 Institute of Health Policy, Management and Evaluation, Faculty of Medicine, University of Toronto, Toronto, Canada; 4 Toronto Health Economics and Technology Assessment Collaborative, University of Toronto, Toronto, Canada

**Keywords:** Childhood obesity, Child growth, Weight, BMI (Body mass Index)

## Abstract

**Background:**

Obesity, with its rising prevalence among Canadians and its estimated cost of $2 billion annually in Canada, is no longer considered a cosmetic issue, but a major health issue that imposes a great burden on the healthcare system and economy. This cross sectional study aims to evaluate the feasibility of identifying the weight status of 6 to 11 year-old children in a university dental clinic using a simplified overweight screening instrument.

**Methods:**

One hundred sixty eight healthy children were enrolled. Weight and height were measured and overweight/obesity status was assessed using two techniques: 1) the 2007 World Health Organization Body Mass Index (BMI)-for-age reference Tables, 2) simplified overweight screening instrument without BMI calculation. Measures of overall, positive, and negative percent agreement between the two approaches were computed.

**Results:**

The children’s average weight, height, BMI and BMI z-score were respectively 32.6 ± 9.5 kg, 133.8 ± 10.7 cm, 17.8 ± 3.2, and 0.4 ± 1.0. The overall, positive, and negative percent agreement between the two screening approaches were respectively, 89%, 100%, and 83%.

**Conclusion:**

This study demonstrated the feasibility and parental acceptance of weight, height and BMI measurement in a dental setting and evidence that supports the validity of a new simplified approach to assess children’s weight status without having to compute BMI.

**Trial registration:**

NCT02637752. Registered 18 December 2015.

**Electronic supplementary material:**

The online version of this article (doi:10.1186/s12887-017-0808-x) contains supplementary material, which is available to authorized users.

## Background

The global prevalence of childhood obesity has increased at an alarming rate. In 2014, an estimated 41 million children under 5 years of age were affected by overweight or obesity, of which, almost three quarters are living in developing countries [1]. Hence, obesity is no longer considered a cosmetic issue, but one of the most serious public health challenges of the 21^st^ century [2], imposing a great burden on the health care system and economy [3]. Therefore, screening children and identifying their overweight and obese status is important [4] as it leads to assessment of the associated comorbidities that occur at an earlier age and progress into adulthood [5]. For example, both Canadian and U.S. clinical practice guidelines recommend that clinicians screen children at age six and older for overweight/obesity [3, 6, 7], and that for such children, weight-related diet and exercise counselling, provided by a multidisciplinary team, should be increased [6, 8].

The most common and practical method to assess obesity in children and adolescents is the Body Mass Index (BMI), the ratio of weight in kilograms to the square of height in meters [9, 10]. Obesity and overweight are defined through BMI-for age percentile plotting, with BMI from 85^th^ to the 94^th^ percentile considered overweight and BMI at or above 95^th^ percentile considered obese for a specific age and gender [11]. As simple as it sounds, the BMI-for age plotting is only used by half of the U.S. pediatricians and family physicians [12, 13]. Additional undertaking and ‘the cumbersome task’ of computing and assessing BMI from height and weight measurements in primary care settings have also been reported as a barrier to the BMI uptake [14–16]

Another group of health care providers with the potential to screen for childhood obesity is dentists. Given 1) the higher frequency of dental visits compared to medical visits (twice a year vs. once a year, especially during childhood) [12], and 2) the routine measurement of the children’s weight and height to calculate safe dosages of local anesthesia for most conscious sedation procedures or dental rehabilitation under general anesthesia, dentists, specifically pediatric dentists, have the potential to identify, assess and refer patients to appropriate resources [14]. Yet, only less than 5% of U.S. pediatric and general dentists offer a form of obesity-related services [14], because mostly they lack training and knowledge for BMI computation and interpretation and guidelines [14, 17].

Recently, a simplified overweight screening instrument was developed based on data derived from the WHO 2007 Growth Reference that would use only the child's height and weight measurements without any additional computation. This simplified screening instrument was based on age- and gender-specific +1 standard deviation z-scores for the BMI, calculated for the various height percentiles at 6-month age intervals (from 5 to 19 years of age), and resulted in two simple tables, one for girls and one for boys that describe overweight screening values for height measurements for all 11 percentiles as described in the 2007 WHO Reference data [18]. With the availability of this simplified approach, our study aims to evaluate the feasibility of identifying the weight status of 6 to 11 year-old children in a university dental clinic setting using either the 2007 WHO BMI-for-age tables or a simplified overweight screening instrument.

## Methods

The cross sectional study refers to the baseline data of our recent randomized controlled clinical trial [18], conducted at the University of Toronto, Faculty of Dentistry from September 2011 to April 2014 (Trial Registration No. NCT02637752). The research protocol and its written informed consent were approved by the Research Ethics Board of the University of Toronto (Protocol No. 28052). The methodology was described in detail previously [19].

**Fig. 1:**
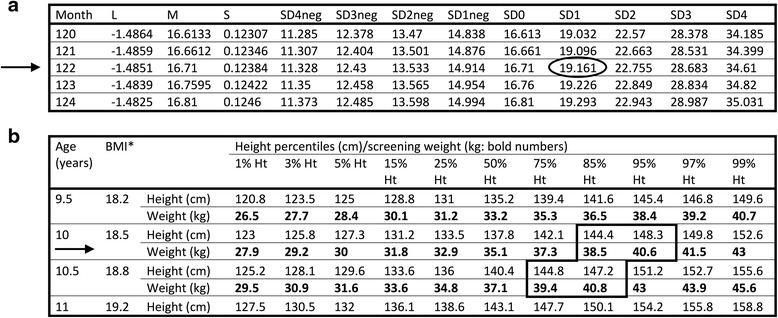
Example of assessing obesity status for a 10 years 2 month old boy, (Height:146 cm; Weight: 38 kg) with or without BMI calculation. **a**) Assessing obesity using WHO 2007 reference tables with BMI calculation. **b**) Assessing obesity using the simplified screening tool without BMI calculation. **a**) In the age row of 122 months, the BMI value for being overweight should be more than +1SD Z-score (19.16). This boy’s computed BMI (38 kg/1.462^2^ m = 17.8) is less than his screening unit grid of 19.161 and hence, he is not overweight. **b**) The age of this boy is between 10 and 10.5 years rows. In the row 10 years, the height is between 144.4 cm and 148.3 cm and in the row 10.5 years, his height is between 144.8 cm and 147.2 cm. The screening weight grids for these heights are, respectively, 38.5, 40.6, 39.4, 40.8 kg. The boy’s weight (38 kg) is below his screening unit grids and hence, he is not overweight. If his weight was 38.7 kg, he would be placed within the screening unit grids and hence, he would have been considered overweight. In contrary, as per the 2007 WHO table, his computed BMI (37.8 kg/1.462^2^ m = 18.15) is still below his screening unit grid of 19.161 and hence, he would have been considered not overweight

In brief, the study population consisted of 168 healthy children (6 to 11 years of age) who attended the undergraduate pediatric dentistry clinic for their routine dental care at the University of Toronto’s Faculty of Dentistry. Upon enrolment at the initial dental visit, the children were assigned to dental students in the undergraduate or graduate pediatric clinic for their required clinical care. The caregivers completed a questionnaire about their sociodemographic characteristics, water and soft drink consumption, school nutrition policy, sedentary and physical activities and video screen time. The questionnaire was adapted mostly from the Canadian Health Measurement Survey [20] as well as other validated Canadian sources [21, 22]. During this time, the research investigator, not involved in the children’s clinical care, measured the children’s weight and height. For the weight measurement, an accurate and calibrated electronic scale (Model 500KL, Health O meter®, USA) was used to weigh the participants [23]. The weight was measured to the nearest 0.1 kg with the child wearing lightweight outer clothing and standing unassisted without shoes on the center of the scale. After repositioning, the measurement was repeated. The two measurements had to agree within 0.1 kg; otherwise, the child’s weight was measured for a third time and the average of the two closest measures were then recorded [24]. For the height measurement, the child was asked to remove hair ornaments, buns, braids, etc. and stand against a calibrated stadiometer without shoes, heels together, legs straight, arms at their sides and shoulders relaxed. The horizontal headpiece of the stadiometer was brought in contact with the top part of the head. The child was instructed to look forward and inhale deeply without raising the heels off the footplate of the stadiometer. The stadiometer was read to the nearest 0.1 cm with the eyes of the individual taking the measurement parallel with the headpiece. The children’s overweight/obesity status were then assessed using two techniques:

1. With BMI calculation: After a child’s height and weight were measured, BMI was computed as weight in kg/ (height in centimeters)^2^. The WHO BMI-for-age reference tables were consulted according to the gender of the individual. Then the child’s age was used to determine the tabular row recommended by WHO BMI-for-age reference tables. Any computed BMI value exceeding the ±1SD BMI Z-scores in the screening tabular row of the table was identified as overweight [24].
2. Without BMI calculation: Overweight status was also determined using a simplified screening instrument without any computation of BMI. This instrument, available from http://dx.doi.org/10.1038/oby.2011.159, [18] consists of two tables, one for girls (Additional file 1: Table S1) and one for boys (Additional file 1: Table S2). To ascertain the weight screening unit grid, the child’s age was selected in the tabular row(s) followed by the height for the tabular column(s). A child was identified as overweight if his/her weight exceeded the screening unit grid. An example is shown in Fig. 1.

### Statistical methods

Assuming a prevalence of unhealthy weight of 30% in the recruited sample, and using the goodness of fit tables [25], (with Kappa null value of 0.40, at two-tailed test null value = 0.40) the required sample size of *n* = 85 would satisfy a power of 80%. The sample size was increased to 168 to satisfy the primary outcome of the larger randomized clinical trial as outlined previously [18]. Data were managed and analyzed using SAS 9.2 software (SAS Institute Inc., Cary, NC, USA). Descriptive analyses were performed using Chi-square test or student *t*-test as indicated. A 2x2 table of results comparing the two approaches for assessing overweight/obesity status were constructed and measures of overall, and positive and negative percent agreement were computed [26, 27]. Statistical tests were two-tailed and interpreted at the 5% significant level.

## Results

A total of 168 subjects (mean age 107.6 ± 18.2 months, 52.4% boys) were enrolled in this study. Many of them [Table 1] were either South Asian or Caucasian, were born in developed countries (mainly Canada), and spoke English/French. More than half of their caregivers had a university degree, worked regular daytime schedule shifts, and were low income family (receiving an annual income of ≤ 40,000 Canadian dollars from wages and salaries). Moreover, a majority of caregivers had not received any previous nutrition/physical counselling. However, they were aware of the school nutrition policy on nutrition standards for food and beverages. A majority of caregivers provided food for their child to take to school or allowed their child to buy nutritious food at school.

**Table 1:** Descriptive characteristics of the study participants

	Variables	Total
*Children*	*Female* (*%*)	47.6
	*Age in months* (*mean ± SD*)	107.6 ± 18.2
	*Racial or cultural groups* (%)	
	South Asian	22.6
	White	21.4
	Black	19.2
	Latin American	8.9
	Southeast Asian	7.7
	Asian	6.5
	Arab	6
	West Asian	4.2
	Mixed	1.8
	Native	0.6
	Guyana	0.6
	Missing	0.6
	*Not aboriginal* (*%*)	98.2
	*Born in a developed country* (*%*)	74.4
	*Speaking English/French* (*%*)	88.1
*Caregivers*	*Highest degree of education* (%)	
	University degree	54.8
	No university degree or diploma	16.7
	Trade certificate or diploma	13.1
	No post-secondary degree, diploma	12.5
	Missing	3
	*Hours at work* (%)	
	Regular day, evening, or night time	59.5
	Rotating shift	15.5
	Not working	19.6
	Missing	5.4
	*Annual income ≤ 40000* (*%*)	64.3
	*Source of income* (%)	
	Wages and salaries	50
	Self-employment	22
	Employment insurance	3.6
	Canada pension plan	1.2
	Child tax benefit	5.4
	Welfare	8.9
	Missing	8.9
	*Nutrition knowledge* (*%*)	
	No previous nutrition counselling	90.5
	Aware of Ontario school nutrition policy	61.3
	Provided food for school	95.8
	Allowed children to buy food at school- knowing the choices there are nutritious	60.1

Table 2 shows the height and weight measurement of the children based on their age (6–8 years and 9–11 years) and gender subgroups. The children had an average weight of 32.6 ± 9.5 kg and an average height of 133.8 ± 10.7 cm. Their mean BMI and BMI z-score were respectively 17.8 ± 3.2 and 0.4 ± 1.0. No significant differences were noted among the participants stratified based on gender and age (*P* > 0.05).

**Table 2:** Baseline measurement (Mean ± SD) for 6–11 year old children enrolled in the study

	Boys 6–8 years old (*n* = 48)	Girls 6–8 years old (*n* = 36)	*P*-value^*^	Boys 9–11 years old (*n* = 40)	Girls 9–11 years old (*n* = 44)	*P*-value*	Total (*N* = 168)
Age (months)	91.2 ± 9.7	93.4 ± 10.2	0.31	122.0 ± 9.0	124.0 ± 9.5	0.34	107.6 ± 18.2
Weight (kg)	27.1 ± 5.4	28.2 ± 7.1	0.41	35.7 ± 8.6	39.4 ± 10.0	0.07	32.6 ± 9.4
Height (cm)	125.4 ± 6.1	127.5 ± 6.8	0.14	139.6 ± 8.0	143.0 ± 8.9	0.07	133.8 ± 10.7
BMI (kg/m^2)^	17.1 ± 2.5	17.1 ± 2.9	0.93	18.1 ± 3.3	19.0 ± 3.7	0.25	17.8 ± 3.2
BMI z-score	0.5 ± 0.9	0.3 ± 1.0	0.58	0.2 ± 1.1	0.4 ± 1.0	0.5	0.4 ± 1.0

* Independent *t* test for differences according to gender

Table 3 shows the weight status comparison by using WHO’s 2007 BMI-for-age tables vs. the simplified overweight screening instrument. Using the simplified screening tables, an overall percent agreement of 89% was achieved between the two methods for detecting weight status. In particular, using the WHO 2007 BMI tables 64.9% of children had a healthy weight status, and 54.1% were identified as healthy by the simplified overweight screening instrument (hence; a negative percent agreement of 83%). The simplified overweight screening instrument had a positive percent agreement of 100% with the WHO’s 2007 BMI-for-age tables in detecting overweight children.

**Table 3:** Weight status comparison using the WHO’s 2007 BMI-for-age tables vs. simplified overweight screening instrument

Weight status comparison		WHO’s 2007 BMI-for-age tables		
		Unhealthy n (%)	Healthy n (%)	Total n (%)
Simplified Screening	Unhealthy n (%)	59 (35.11) (a)	18 (10.71) (b)	77 (45.83)
	Healthy n (%)	0 (0) (c)	91 (54.16) (d)	91 (54.16)
	Total n (%)	59 (35.11)	109 (64.88)	168 (100)

This table shows the weight status comparison by using WHO’s 2007 BMI-for-age tables vs. the study simplified overweight screening instrument

Overall percent agreement = 100% x (a + d)/(a + b + c + d) = 100% x 150/168 = 89%

Positive percent agreement = 100% x a/(a + c) = 100% x 59/59 = 100%

Negative percent agreement = 100% x d/(b + d) = 100% x 91/109 = 83%

## Discussion

Childhood obesity is a much bigger concern than its obvious impact on physical health. Obese children have a lower quality of life and impaired social functioning [28], and are at an increased risk of many conditions including: type 2 diabetes, hypertension, dyslipidemia, elevated cholesterol, coronary artery disease, obstructive sleep apnea, stroke, fatty liver disease, osteoarthritis, orthopedic problems and certain forms of cancer [3, 29]. The objective of this study was to identify the weight status of children from 6 to 11 years old in a dental setting and to evaluate the agreement between the WHO BMI-for-age tables and the simplified screening instrument tables, which were developed based on height and weight measurements and are derived from the 2007 WHO reference tables [18].

The BMI is the best method to identify obesity/overweight in children and depends on accurate weighing and measuring and making correct clinical judgments on the appropriateness of a child’s pattern of growth, for most clinical, screening and surveillance purposes [30]. However, its under-utilization by healthcare providers, in particular in primary care setting, has been a point of concern [12–17, 31]. While it can be argued that the use of online BMI calculator and electronic health record and their integration to the clinical practice would enhance the use of BMI and the adherence to recommendations for screening and identification of childhood overweight and obesity [32]; some barriers such as navigation pathway needed to locate the required obesity-related form in the electronic health record, and minimal training of physicians, consistent with those encountered in other electronic health record interventions [33], remains to be the challenges associated with implementing interventions in a complex care system [34]. Furthermore, poor information and communication technology, a limited to no access to internet, defective power resource, costs of equipment supply, and low level of technical skills can all play as barriers for health care providers in developing countries where the most burden of the disease is expected [35].

The simplified overweight screening instrument utilized in this study was previously published [18] and was developed following the methodology of Kaelber and Pickett in developing their simplified screening tool to identify children and adolescents needing further evaluation of blood pressure [36]. This simplified tool consists of two gender-specific tables, through which, with the use of only the child's height and weight measurements without any additional computation, overweight screening can be performed.

There has been an increasing trends in visitation patterns of children to dentists [12], with a higher frequency of annual visits as compared to medical visits [12, 37, 38]. Children’s weight and height data are being routinely collected in dental offices who care for children, as part of a new patient medical history evaluation, and for calculation of safe dosages of local anesthesia for dental treatment under conscious or deep sedation. Therefore, dentists, specially pediatric dentists, can utilize dental visits to add additional screening and counseling that complements physicians’ efforts in addressing overweight or obesity and to refer those with unhealthy weight status to pediatricians or family physicians for further evaluations [14, 39]. In our setting, we found the application of this tool to be very feasible: with the minimal needed equipment, we were able to gather the weight and height from 168 children and use the tool to screen for overweight with no disruption to patient flow.

Our results showed an 89% overall agreement between the WHO BMI-for-age reference tables and the simplified screening instrument utilized in this research. In particular, the positive percent agreement of 100% means that none of the unhealthy weight individuals who were reported by WHO tables were screened as healthy weight status according to the screening tables. The only difference between the two techniques is that the simplified approach identified 18 participants as unhealthy weight status, while these participants were judged as healthy based on the WHO tables. This is in particular for the cases whose weight/height values put them on the borderline of being overweight. An example is illustrated in Fig. 1b. It can be argued that this difference is not only worrying, but also, could be a benefit for early identification of children with borderline unhealthy weight status due to the higher sensitivity of the simplified screening tables.

Although the study population was limited to a convenient university-based sample, for the purpose of the study objective, our study sample has an acceptable generalizability since 64.9% of our subjects, as compared to 65.5% of Canadian children from the Canadian Health Measurement Survey [40], had healthy weight status (*P* = 0.72). Hence, based on the generalizability and validity of this simplified screening instrument, it is expected that this screening tool would remove the burden of BMI calculation and would hopefully enhance overweight triage in primary care settings as well as school-based and community surveillance efforts [18]. It has to be acknowledged that regardless, the healthcare professionals, specially those who care for children, would still need to have access to appropriate equipment to measure weight and height, introduce the idea of taking these measurements then undertake these measurements. At the end, health care professionals should promote active healthy living within each family unit, with focus on health rather than the actual weight numbers or physical appearance [41]. An important aspect of the clinician’s responsibility that remains is related to communicating the overweight screening results to the parent in a supportive and culturally appropriate way rather than an accusatory way [42], in a manner that avoids judgment and the instillation of guilt in the parents [39].

## Conclusions

This study has demonstrated the feasibility of weight, height and BMI measurement in a dental setting. It has further shown evidence that supports validity of a new simplified approach to assess children’s weight status without having to compute BMI. This simplified screening can enhance overweight triage in primary care settings as well as school-based and community surveillance efforts. Dentists who collaborate with other health care professionals have the potential to address childhood overweight/obesity and should determine height, weight for their patients at least annually and refer patients with unhealthy weight status to pediatricians, family physicians or registered dieticians.

## Additional file

Additional file 1: Screening weights for overweight in girls and boys. Screening weights for overweight in girls (**Table S1.**) and boys (**Table S2.**) based on age and 2007 WHO Reference height percentiles and BMI. (PDF 1276 kb)

## Abbreviations

BMI: Body mass index
WHO: World Health Organization.
