# Supplementary material for: Validity of a simplified screening instrument for assessing overweight children in a dental setting: a cross sectional study
Source: BMC Pediatr. 2017 Feb 17;17:56. doi: 10.1186/s12887-017-0808-x (PMC5316148; doi:10.1186/s12887-017-0808-x)
Supplement: Additional file 1: — Screening weights for overweight in girls and boys. Screening weights for overweight in girls (Table S1.) and boys (Table S2.) based on age and 2007 WHO Reference height percentiles and BMI. (PDF 1276 kb) [file 12887_2017_808_MOESM1_ESM.pdf]

Table S1 Screening weights for overweight in girls based on age and 2007 WHO Reference height percentiles and BMI

Age (years)	BMI (z-scores + 1 SD)		Height percentiles (cm)/ screening weight (kg; bold numbers)										
			1% Ht	3% Ht	5% Ht	15% Ht	25% Ht	50% Ht	75% Ht	85% Ht	95% Ht	97% Ht	99% Ht
5*	16.9	Ht (cm)	98.5	100.6	101.8	104.7	106.4	109.6	112.8	114.5	117.5	118.6	120.7
		Wt (kg)	**16.4**	**17.1**	**17.5**	**18.5**	**19.1**	**20.3**	**21.5**	**22.1**	**23.3**	**23.7**	**24.6**
5.5	16.9	Ht (cm)	100.7	102.9	104.1	107.1	108.8	112.2	115.5	117.3	120.3	121.5	123.7
		Wt (kg)	**17.2**	**17.9**	**18.3**	**19.4**	**20.1**	**21.3**	**22.6**	**23.3**	**24.5**	**25**	**25.9**
6	17	Ht (cm)	103.2	105.5	106.7	109.8	111.7	115.1	118.6	120.4	123.5	124.8	127
		Wt (kg)	**18.1**	**18.9**	**19.4**	**20.5**	**21.2**	**22.6**	**23.9**	**24.7**	**26**	**26.5**	**27.5**
6.5	17.1	Ht (cm)	105.7	108	109.3	112.5	114.4	118	121.5	123.5	126.7	127.9	130.3
		Wt (kg)	**19.1**	**20**	**20.5**	**21.7**	**22.4**	**23.8**	**25.3**	**26.1**	**27.5**	**28**	**29.1**
7	17.3	Ht (cm)	108.1	110.5	111.8	115.1	117.1	120.8	124.5	126.5	129.8	131.1	133.5
		Wt (kg)	**20.2**	**21.1**	**21.6**	**22.9**	**23.7**	**25.2**	**26.8**	**27.7**	**29.1**	**29.7**	**30.8**
7.5	17.5	Ht (cm)	110.6	113.1	114.4	117.8	119.9	123.7	127.5	129.5	132.9	134.3	136.8
		Wt (kg)	**21.4**	**22.4**	**22.9**	**24.3**	**25.1**	**26.7**	**28.4**	**29.3**	**30.9**	**31.5**	**32.7**
8	17.7	Ht (cm)	113.1	115.7	117	120.5	122.6	126.6	130.5	132.6	136.1	137.5	140
		Wt (kg)	**22.7**	**23.7**	**24.3**	**25.8**	**26.7**	**28.4**	**30.2**	**31.2**	**32.8**	**33.5**	**34.8**
8.5	18	Ht (cm)	115.6	118.3	119.7	123.3	125.5	129.5	133.5	135.7	139.3	140.7	143.4
		Wt (kg)	**24.1**	**25.2**	**25.8**	**27.4**	**28.4**	**30.2**	**32.1**	**33.2**	**35**	**35.7**	**37**
9	18.3	Ht (cm)	118.3	121	122.4	126.2	128.4	132.5	136.6	138.8	142.5	144	146.7
		Wt (kg)	**25.6**	**26.8**	**27.5**	**29.2**	**30.2**	**32.2**	**34.2**	**35.3**	**37.2**	**38**	**39.4**
9.5	18.7	Ht (cm)	121	123.8	125.2	129.1	131.3	135.5	139.8	142	145.8	147.3	150.1
		Wt (kg)	**27.3**	**28.6**	**29.3**	**31.1**	**32.2**	**34.3**	**36.5**	**37.7**	**39.7**	**40.5**	**42.1**
10	19	Ht (cm)	123.8	126.6	128.1	132	134.3	138.6	143	145.3	149.2	150.7	153.5
		Wt (kg)	**29.2**	**30.5**	**31.2**	**33.2**	**34.3**	**36.6**	**38.9**	**40.2**	**42.3**	**43.2**	**44.9**
10.5	19.4	Ht (cm)	126.6	129.5	131.1	135	137.4	141.8	146.2	148.6	152.5	154.1	157
		Wt (kg)	**31.1**	**32.6**	**33.4**	**35.4**	**36.7**	**39.1**	**41.5**	**42.9**	**45.2**	**46.1**	**47.9**
11	19.9	Ht (cm)	129.5	132.5	134.1	138.1	140.5	145	149.5	151.9	155.9	157.5	160.5
		Wt (kg)	**33.3**	**34.9**	**35.7**	**37.9**	**39.2**	**41.8**	**44.4**	**45.8**	**48.3**	**49.3**	**51.1**
11.5	20.3	Ht (cm)	132.5	135.5	137.1	141.2	143.6	148.2	152.7	155.2	159.3	160.9	163.9
		Wt (kg)	**35.7**	**37.3**	**38.2**	**40.5**	**41.9**	**44.6**	**47.4**	**48.9**	**51.6**	**52.6**	**54.6**
12	20.8	Ht (cm)	135.3	138.4	140	144.1	146.6	151.2	155.8	158.3	162.5	164.1	167.1
		Wt (kg)	**38.1**	**39.8**	**40.8**	**43.2**	**44.7**	**47.6**	**50.5**	**52.2**	**54.9**	**56**	**58.1**
12.5	21.3	Ht (cm)	137.9	141	142.6	146.8	149.3	154	158.7	161.2	165.4	167	170.1
		Wt (kg)	**40.5**	**42.4**	**43.4**	**45.9**	**47.5**	**50.5**	**53.6**	**55.3**	**58.3**	**59.4**	**61.6**

Age (years)	BMI (z-scores + 1 SD)		Height percentiles (cm)/ screening weight (kg; bold numbers)										
			1% Ht	3% Ht	5% Ht	15% Ht	25% Ht	50% Ht	75% Ht	85% Ht	95% Ht	97% Ht	99% Ht
13	21.8	Ht (cm)	140.2	143.3	145	149.2	151.7	156.4	161.1	163.6	167.8	169.4	172.5
		Wt (kg)	**42.9**	**44.8**	**45.8**	**48.5**	**50.2**	**53.3**	**56.6**	**58.3**	**61.4**	**62.6**	**64.9**
13.5	22.3	Ht (cm)	142.1	145.2	146.9	151.1	153.6	158.3	163	165.5	169.7	171.4	174.5
		Wt (kg)	**45**	**47**	**48.1**	**50.9**	**52.6**	**55.8**	**59.2**	**61**	**64.2**	**65.4**	**67.8**
14	22.7	Ht (cm)	143.6	146.7	148.4	152.6	155.1	159.8	164.5	167	171.2	172.8	175.9
		Wt (kg)	**46.9**	**48.9**	**50**	**52.9**	**54.7**	**58**	**61.5**	**63.4**	**66.6**	**67.9**	**70.4**
14.5	23.1	Ht (cm)	144.8	147.9	149.5	153.7	156.2	160.9	165.6	168.1	172.3	173.9	177
		Wt (kg)	**48.5**	**50.6**	**51.7**	**54.7**	**56.5**	**59.9**	**63.4**	**65.4**	**68.7**	**70**	**72.5**
15	23.5	Ht (cm)	145.7	148.7	150.4	154.5	157	161.7	166.3	168.8	173	174.6	177.7
		Wt (kg)	**49.9**	**52**	**53.2**	**56.2**	**58**	**61.5**	**65**	**67**	**70.4**	**71.7**	**74.2**
15.5	23.8	Ht (cm)	146.3	149.3	150.9	155.1	157.6	162.2	166.8	169.3	173.4	175	178.1
		Wt (kg)	**51**	**53.2**	**54.3**	**57.3**	**59.2**	**62.7**	**66.3**	**68.3**	**71.7**	**73**	**75.6**
16	24.1	Ht (cm)	146.7	149.8	151.4	155.5	157.9	162.5	167.1	169.6	173.7	175.3	178.3
		Wt (kg)	51.9	54.1	55.2	58.3	60.1	63.7	67.3	69.3	72.7	74.1	76.6
16.5	24.3	Ht (cm)	147	150	151.6	155.7	158.2	162.7	167.3	169.7	173.8	175.4	178.4
		Wt (kg)	**52.6**	**54.8**	**55.9**	**59**	**60.9**	**64.4**	**68.1**	**70.1**	**73.5**	**74.8**	**77.4**
17	24.5	Ht (cm)	147.3	150.3	151.8	155.9	158.3	162.9	167.4	169.8	173.9	175.4	178.4
		Wt (kg)	**53.2**	**55.3**	**56.5**	**59.6**	**61.4**	**65**	**68.6**	**70.6**	**74.1**	**75.4**	**78**
17.5	24.6	Ht (cm)	147.5	150.5	152	156.1	158.5	163	167.5	169.9	173.9	175.5	178.4
		Wt (kg)	**53.6**	**55.8**	**57**	**60**	**61.9**	**65.5**	**69.1**	**71.1**	**74.5**	**75.9**	**78.5**
18	24.8	Ht (cm)	147.7	150.6	152.2	156.2	158.6	163.1	167.5	169.9	173.9	175.5	178.4
		Wt (kg)	**54**	**56.2**	**57.4**	**60.4**	**62.3**	**65.9**	**69.5**	**71.5**	**74.9**	**76.3**	**78.9**
18.5	24.9	Ht (cm)	147.8	150.8	152.3	156.3	158.7	163.1	167.6	169.9	173.9	175.5	178.4
		Wt (kg)	**54.4**	**56.5**	**57.7**	**60.8**	**62.6**	**66.2**	**69.8**	**71.8**	**75.3**	**76.6**	**79.2**
19	25	Ht (cm)	147.9	150.9	152.4	156.4	158.7	163.2	167.6	169.9	173.9	175.5	178.4
		Wt (kg)	**54.6**	**56.8**	**58**	**61.1**	**62.9**	**66.5**	**70.1**	**72.1**	**75.5**	**76.9**	**79.4**

*Values of BMI and height are for 61 months of age. Ht: Height; Wt: Weight, SD: Standard Deviation;

The highlighted cells present an assessment for a 6-year-8-month-old girl, whose height and weight are, respectively, 124 cm and 29 kg.
Step 1: This girl's age (6 years and 8 months) is between the rows 6.5 years and 7 years.
Step 2: In the row 6.5 years, the girl's height (124cm) is between the columns 123.5 cm and 126.7 cm.
Step 3: The screening weight for 126.7 cm is 27.5 kg.
Step 4: The girl's weight (29kg) is more this screening unit grid of 27.5 kg. Therefore; this girl is overweight

Note: Computation of individual BMI will be required for cases with height/weight values that occur between tabular values with inability for clear delineation.

Table S2 Screening weights for overweight in boys based upon age and 2007 WHO Reference height percentiles and BMI

Age (years)	BMI (z-scores + 1 SD)		Height percentiles (cm)/ screening weight (kg; bold numbers)										
			1% Ht	3% Ht	5% Ht	15% Ht	25% Ht	50% Ht	75% Ht	85% Ht	95% Ht	97% Ht	99% Ht
5*	16.6	Ht (cm)	99.6	101.6	102.7	105.5	107.2	110.3	113.4	115	117.8	118.9	120.9
		Wt (kg)	**16.5**	**17.2**	**17.6**	**18.5**	**19.1**	**20.2**	**21.4**	**22**	**23.1**	**23.5**	**24.4**
5.5	16.7	Ht (cm)	101.9	104	105.1	108	109.7	112.9	116.1	117.8	120.7	121.8	124
		Wt (kg)	**17.3**	**18**	**18.4**	**19.5**	**20.1**	**21.3**	**22.5**	**23.2**	**24.3**	**24.8**	**25.6**
6	16.8	Ht (cm)	104.5	106.7	107.8	110.8	112.6	116	119.3	121.1	124.1	125.2	127.4
		Wt (kg)	**18.3**	**19.1**	**19.5**	**20.6**	**21.3**	**22.5**	**23.8**	**24.6**	**25.8**	**26.3**	**27.2**
6.5	16.9	Ht (cm)	107	109.3	110.5	113.6	115.4	118.9	122.3	124.2	127.3	128.5	130.7
		Wt (kg)	**19.3**	**20.2**	**20.6**	**21.8**	**22.5**	**23.9**	**25.3**	**26**	**27.4**	**27.9**	**28.9**
7	17	Ht (cm)	109.4	111.8	113	116.3	118.2	121.7	125.3	127.2	130.4	131.7	134
		Wt (kg)	**20.4**	**21.3**	**21.8**	**23**	**23.8**	**25.3**	**26.8**	**27.6**	**29**	**29.6**	**30.6**
7.5	17.2	Ht (cm)	111.8	114.3	115.5	118.9	120.8	124.5	128.2	130.2	133.5	134.8	137.3
		Wt (kg)	**21.5**	**22.5**	**23**	**24.4**	**25.2**	**26.7**	**28.3**	**29.2**	**30.7**	**31.3**	**32.5**
8	17.4	Ht (cm)	114.1	116.6	118	121.4	123.5	127.3	131.1	133.1	136.6	137.9	140.4
		Wt (kg)	**22.7**	**23.7**	**24.3**	**25.7**	**26.6**	**28.2**	**30**	**30.9**	**32.5**	**33.2**	**34.4**
8.5	17.7	Ht (cm)	116.4	119	120.3	123.9	126	129.9	133.9	136	139.5	140.9	143.5
		Wt (kg)	**23.9**	**25**	**25.6**	**27.1**	**28**	**29.8**	**31.7**	**32.7**	**34.4**	**35.1**	**36.4**
9	17.9	Ht (cm)	118.6	121.3	122.7	126.3	128.5	132.6	136.6	138.8	142.5	143.9	146.6
		Wt (kg)	**25.2**	**26.3**	**27**	**28.6**	**29.6**	**31.5**	**33.4**	**34.5**	**36.3**	**37.1**	**38.5**
9.5	18.2	Ht (cm)	120.8	123.5	125	128.8	131	135.2	139.4	141.6	145.4	146.8	149.6
		Wt (kg)	**26.5**	**27.7**	**28.4**	**30.1**	**31.2**	**33.2**	**35.3**	**36.5**	**38.4**	**39.2**	**40.7**
10	18.5	Ht (cm)	123	125.8	127.3	131.2	133.5	137.8	142.1	144.4	148.3	149.8	152.6
		Wt (kg)	**27.9**	**29.2**	**30**	**31.8**	**32.9**	**35.1**	**37.3**	**38.5**	**40.6**	**41.5**	**43**
10.5	18.8	Ht (cm)	125.2	128.1	129.6	133.6	136	140.4	144.8	147.2	151.2	152.7	155.6
		Wt (kg)	**29.5**	**30.9**	**31.6**	**33.6**	**34.8**	**37.1**	**39.4**	**40.8**	**43**	**43.9**	**45.6**
11	19.2	Ht (cm)	127.5	130.5	132	136.1	138.6	143.1	147.7	150.1	154.2	155.8	158.8
		Wt (kg)	**31.1**	**32.6**	**33.4**	**35.5**	**36.8**	**39.3**	**41.8**	**43.2**	**45.6**	**46.5**	**48.3**
11.5	19.5	Ht (cm)	129.9	133	134.6	138.8	141.3	146	150.6	153.1	157.4	159	162.1
		Wt (kg)	**33**	**34.6**	**35.4**	**37.7**	**39**	**41.7**	**44.4**	**45.8**	**48.4**	**49.4**	**51.3**
12	19.9	Ht (cm)	132.6	135.8	137.4	141.7	144.3	149.1	153.9	156.4	160.7	162.4	165.6
		Wt (kg)	**35.1**	**36.8**	**37.7**	**40.1**	**41.5**	**44.3**	**47.2**	**48.8**	**51.5**	**52.6**	**54.7**
12.5	20.4	Ht (cm)	135.6	138.8	140.5	144.9	147.5	152.4	157.3	160	164.4	166.1	169.3
		Wt (kg)	**37.4**	**39.3**	**40.2**	**42.8**	**44.4**	**47.4**	**50.4**	**52.1**	**55.1**	**56.2**	**58.4**
13	20.8	Ht (cm)	138.8	142.1	143.8	148.3	151	156	161.1	163.7	168.3	170	173.3
		Wt (kg)	**40.1**	**42**	**43.1**	**45.8**	**47.5**	**50.7**	**54**	**55.8**	**59**	**60.2**	**62.6**

Age (years)	BMI (z-scores + 1 SD)		Height percentiles (cm)/ screening weight (kg; bold numbers)										
			1% Ht	3% Ht	5% Ht	15% Ht	25% Ht	50% Ht	75% Ht	85% Ht	95% Ht	97% Ht	99% Ht
13.5	21.3	Ht (cm)	142.1	145.4	147.2	151.8	154.6	159.7	164.8	167.5	172.2	173.9	177.3
		Wt (kg)	**43**	**45.1**	**46.2**	**49.1**	**50.9**	**54.3**	**57.9**	**59.8**	**63.1**	**64.4**	**67**
14	21.8	Ht (cm)	145.3	148.7	150.5	155.2	158	163.2	168.4	171.2	175.8	177.6	181.1
		Wt (kg)	**46**	**48.2**	**49.3**	**52.4**	**54.3**	**58**	**61.7**	**63.8**	**67.3**	**68.7**	**71.4**
14.5	22.2	Ht (cm)	148.2	151.7	153.5	158.3	161.1	166.3	171.5	174.4	179.1	180.9	184.4
		Wt (kg)	**48.9**	**51.2**	**52.4**	**55.7**	**57.7**	**61.5**	**65.4**	**67.6**	**71.3**	**72.8**	**75.6**
15	22.7	Ht (cm)	150.8	154.3	156.1	160.9	163.7	169	174.2	177	181.8	183.6	187.1
		Wt (kg)	**51.6**	**54**	**55.3**	**58.7**	**60.8**	**64.8**	**68.9**	**71.1**	**75**	**76.5**	**79.4**
15.5	23.1	Ht (cm)	153	156.5	158.3	163.1	165.9	171.1	176.4	179.2	184	185.8	189.3
		Wt (kg)	**54.1**	**56.6**	**57.9**	**61.5**	**63.6**	**67.7**	**71.9**	**74.3**	**78.3**	**79.8**	**82.8**
16	23.5	Ht (cm)	154.8	158.3	160.1	164.8	167.7	172.9	178.1	181	185.7	187.5	191
		Wt (kg)	**56.4**	**58.9**	**60.3**	**63.9**	**66.1**	**70.3**	**74.7**	**77**	**81.1**	**82.7**	**85.8**
16.5	23.9	Ht (cm)	156.3	159.7	161.5	166.2	169	174.2	179.4	182.2	186.9	188.7	192.2
		Wt (kg)	**58.4**	**61**	**62.4**	**66.1**	**68.3**	**72.6**	**77**	**79.4**	**83.5**	**85.2**	**88.3**
17	24.3	Ht (cm)	157.4	160.8	162.6	167.2	170	175.2	180.3	183.1	187.7	189.5	192.9
		Wt (kg)	**60.1**	**62.7**	**64.2**	**67.9**	**70.1**	**74.5**	**78.9**	**81.4**	**85.5**	**87.2**	**90.4**
17.5	24.6	Ht (cm)	158.2	161.5	163.3	167.9	170.7	175.8	180.9	183.6	188.2	190	193.4
		Wt (kg)	**61.6**	**64.2**	**65.6**	**69.4**	**71.7**	**76**	**80.5**	**82.9**	**87.1**	**88.8**	**92**
18	24.9	Ht (cm)	158.8	162.1	163.9	168.4	171.1	176.1	181.2	183.9	188.4	190.2	193.5
		Wt (kg)	**62.8**	**65.5**	**66.9**	**70.7**	**72.9**	**77.3**	**81.8**	**84.2**	**88.5**	**90.1**	**93.3**
18.5	25.2	Ht (cm)	159.2	162.5	164.2	168.7	171.4	176.4	181.4	184	188.5	190.3	193.6
		Wt (kg)	**63.9**	**66.5**	**68**	**71.7**	**74**	**78.4**	**82.9**	**85.3**	**89.5**	**91.2**	**94.4**
19	25.4	Ht (cm)	159.6	162.8	164.5	169	171.6	176.5	181.5	184.1	188.5	190.3	193.5
		Wt (kg)	**64.8**	**67.5**	**68.9**	**72.7**	**75**	**79.3**	**83.8**	**86.3**	**90.5**	**92.1**	**95.3**

*Values of BMI and height are for 61 months of age. Ht: Height; Wt: Weight, SD: Standard Deviation;

The highlighted cells present an assessment for a 9-year-8-month-old boy, whose height and weight are, respectively, 143 cm and 35 kg.
Step 1: This boy's age (9 years and 8 months) is between the rows 9.5 years and 10 years.
Step 2: In the row 9.5 years, the boy's height (143cm) is between the columns 141.6 cm and 145.4 cm.
Step 3: The screening weight for 141.6 cm is 36.5 kg.
Step 4: The boy's weight (35kg) is below this screening unit grid of 36.5 kg. Therefore; this boy is not overweight

Note: Computation of individual BMI will be required for cases with height/weight values that occur between tabular values with inability for clear delineation.
